# The ginsenoside PPD exerts anti-endometriosis effects by suppressing estrogen receptor-mediated inhibition of endometrial stromal cell autophagy and NK cell cytotoxicity

**DOI:** 10.1038/s41419-018-0581-2

**Published:** 2018-05-14

**Authors:** Bing Zhang, Wen-Jie Zhou, Chun-Jie Gu, Ke Wu, Hui-Li Yang, Jie Mei, Jia-Jun Yu, Xiao-Fan Hou, Jian-Song Sun, Feng-Yuan Xu, Da-Jin Li, Li-Ping Jin, Ming-Qing Li

**Affiliations:** 1 Laboratory for Reproductive Immunology, Key Laboratory of Reproduction Regulation of NPFPC, SIPPR, IRD, Hospital of Obstetrics and Gynecology, Fudan University Shanghai Medical College, 200011 Shanghai, China; 2 National Research Centre for Carbohydrate Synthesis, Jiangxi Normal University, 330022 Jiangxi Nanchang, China; 3 Wallace H.Coulter Department of Biomedical Engineering, Georgia Tech College of Engineering and Emory School of Medicine, Georgia Institute of Technology, Atlanta, 30332 GA USA; 4 Clinical and Translational Research Center, Shanghai First Maternity and Infant Hospital, Tongji University School of Medicine, 200040 Shanghai, China; 5 Shanghai Key Laboratory of Female Reproductive Endocrine Related Diseases, 200011 Shanghai, China

## Abstract

Endometriosis (EMS) is an estrogen-dependent gynecological disease with a low autophagy level of ectopic endometrial stromal cells (eESCs). Impaired NK cell cytotoxic activity is involved in the clearance obstruction of the ectopic endometrial tissue in the abdominopelvic cavity. Protopanaxadiol (PPD) and protopanaxatriol (PPT) are two metabolites of ginsenosides, which have profound biological functions, such as anti-cancer activities. However, the role and mechanism of ginsenosides and metabolites in endometriosis are completely unknown. Here, we found that the compounds PPD, PPT, ginsenoside-Rg3 (G-Rg3), ginsenoside-Rh2 (G-Rh2), and esculentoside A (EsA) led to significant decreases in the viability of eESCs, particularly PPD (IC50 = 30.64 µM). In vitro and in vivo experiments showed that PPD promoted the expression of progesterone receptor (PR) and downregulated the expression of estrogen receptor α (ERα) in eESCs. Treatment with PPD obviously induced the autophagy of eESCs and reversed the inhibitory effect of estrogen on eESC autophagy. In addition, eESCs pretreated with PPD enhanced the cytotoxic activity of NK cells in response to eESCs. PPD decreased the numbers and suppressed the growth of ectopic lesions in a mouse EMS model. These results suggest that PPD plays a role in anti-EMS activation, possibly by restricting estrogen-mediated autophagy regulation and enhancing the cytotoxicity of NK cells. This result provides a scientific basis for potential therapeutic strategies to treat EMS by PPD or further structural modification.

## Introduction

Endometriosis (EMS) is a common estrogen-dependent gynecological disease that is defined by the attachment of endometrial tissue at extrauterine ectopic sites where it forms invasive lesions^1–3^. It affects ~5–15% of all women of reproductive age and 20–50% of all infertile women^4,5^. There is strong evidence to support the important role of estrogen dependence, progesterone resistance, and defects in the immune system (e.g., impaired cytotoxic activity of NK cells) in EMS, leading to the enhanced benign tumor-like behaviors of refluxed endometrium (such as unrestrained proliferation and aggressive invasion)^6–9^.

Current medical therapies (e.g., progestins, androgens, gonadotropin-releasing hormone (GnRH) agonists, and aromatase inhibitors) focus primarily on reducing the systemic levels of estrogens, but these are of limited effectiveness, with frequent recurrence and considerable side effects^1^. Therefore, the most urgent need is to better understand the mechanisms underlying EMS, to enable the development of more effective treatments.

As a constitutive catabolic pathway, autophagy is the natural, regulated, destructive mechanism of the cell that mediates both non-specific and targeted sequestration of cellular organelles and other macromolecules, allowing the orderly degradation of cellular components in lysosomes and the recycling of bioenergetics metabolites^10^. Accumulating evidence has indicated that the level of autophagy in both the ectopic and eutopic endometrium of patients with EMS is decreased^7,11–15^. This aberrantly low autophagy is associated with the highly proliferative and lowly apoptotic capacities of ectopic endometrial stromal cells (eESCs)^7,11–14^. Our previous work has showed that estrogen promotes the survival of human ESCs via upregulating CXCL12/CXCR4-mediated autophagy inhibition^7^. The CXCR4-CXCL12 axis has also been shown to have both immune (e.g., lymphocyte chemotaxis, especially for NK cells) and non-immune functions (e.g., tissue repair, angiogenesis, cell invasion, and migration)^16,17^. In addition, our recent study has observed that the low autophagy of ESCs leads to the impaired cytotoxicity of NK cells in a co-culture system, which may be beneficial to the immune escape of ectopic ESCs^8^. Therefore, a new therapeutic strategy should be urgently required, that is, targeting autophagy and immunomodulation simultaneously.

As a traditional medicinal herb, ginseng is widely used in Asian countries and North America. Ginsenosides (e.g., ginsenoside-Rg3 (G-Rg3) and ginsenoside Rh2 (G-Rh2)) are the main components extracted from ginseng and have various pharmaceutical activities, such as antitumor, antioxidant, immunomodulatory, and anti-inflammatory activities^18–21^. As two metabolites of ginsenoside, protopanaxadiol (PPD) and protopanaxatriol (PPT) also exhibit activity against various cancer cells^20,22,23^. Various functional assays and molecular docking studies have provided evidence that ginsenosides can mediate their cellular activities by binding to the active sites of steroid receptors (e.g., estrogen receptor α (ERα))^20^. However, to date, no study has been published regarding the anti-EMS (a benign estrogen-dependent disease with tumor-like behaviors) activities of these ginsenosides and metabolites.

Therefore, the aim of this study was to investigate whether ginsenosides and metabolites have anti-EMS activity, and if so, whether this effect is dependent on the regulation of autophagy and ER in eESCs and the cytotoxicity of NK cells in vitro and in vivo.

## Results

### PPD has powerful anti-EMS activity in vitro

**Fig. 1:**
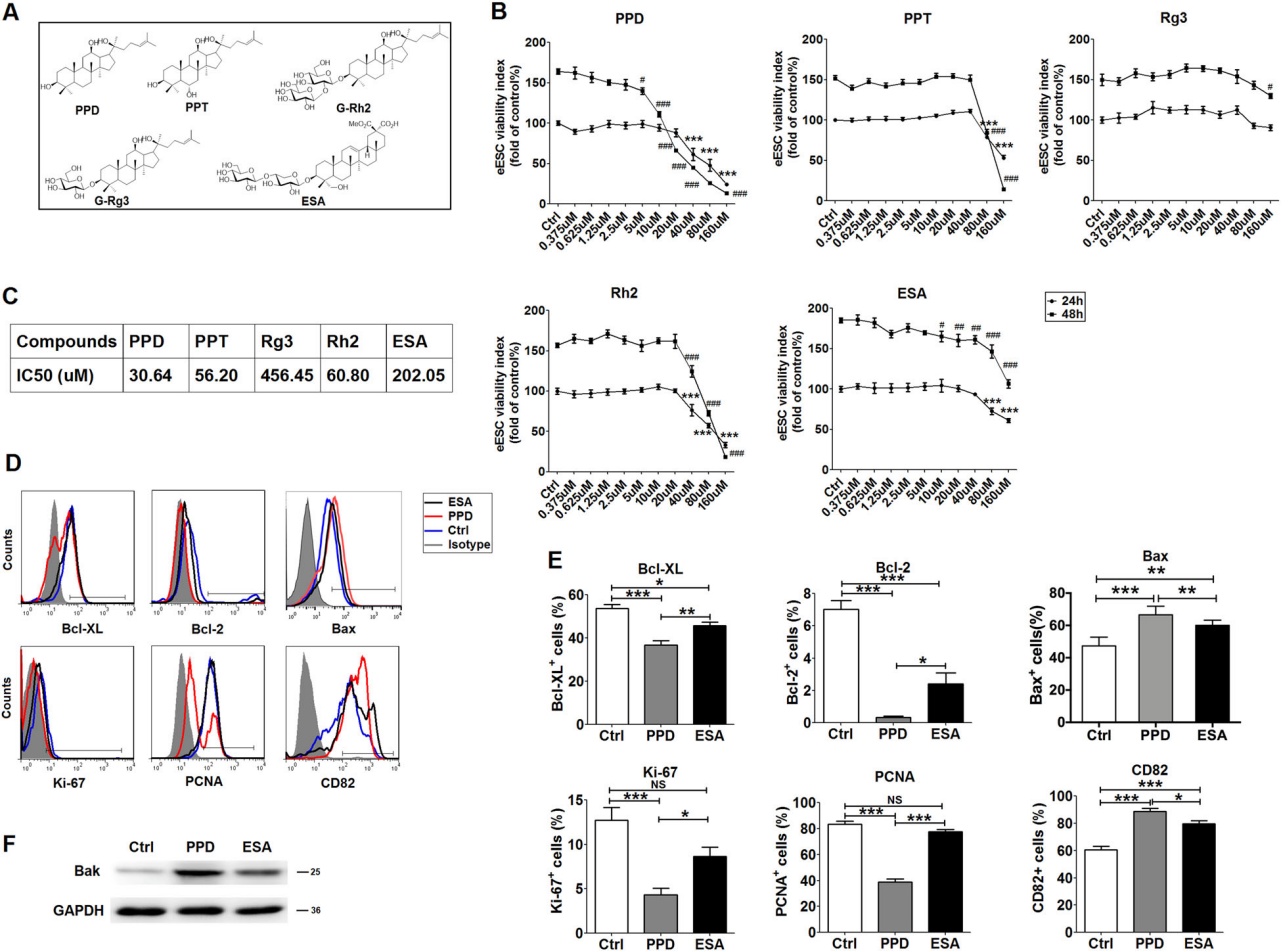
PPD has powerful anti-EMS activity in vitro. **a** Chemical structures of PPD, PPT, G-Rg3, G-Rh2, and EsA. **b** After treatment with PPD, PPT, G-Rg3, G-Rh2 or EsA at different concentrations (0–160 µM) for 24 or 48 h, the viability of ectopic ESCs (eESCs, *n* = 6) was analyzed by the CCK-8 assay (one-way ANOVA). **c** IC50 of the compounds in eESCs. **d**, **e** After treatment with PPD or EsA (40 µM) for 48 h, the expression levels of Bcl-xL, Bcl-2, Bax, Ki-67, PCNA, and CD82 in ectopic ESCs (eESCs, *n* = 6) were analyzed by FCM (one-way ANOVA). **f** After treatment with PPD or EsA (40 µM) for 48 h, the expression levels of Bak in eESCs (*n* = 6) were analyzed by western blotting. Data are expressed as mean ± SEM. *P < 0.05, **P < 0.01, and ***P < 0.001 (compared to ctrl for 24 h); ^#^*P* < 0.05, ^##^*P* < 0.01, and ^##^*P* < 0.001 (compared to ctrl for 48 h). NS no statistically difference

Esculentoside A (EsA), a triterpene saponin isolated from the root of the Chinese herb Phytolacca esculenta, is known for its anti-inflammatory and anti-oxidative effects^24^. It has been reported that EsA inhibits the growth of ectopic lesions in a rat EMS model, and its effect is stronger than that of the same dose of azole (a synthetic steroid that is used primarily in the treatment of EMS) (China Patent Number: CN200510110906.X). The chemical structures of PPD, PPT, G-Rg3, G-Rh2 and EsA are shown in Figure 1a. Owing to the anti-EMS activity and similar chemical structures among EsA, ginsenosides and metabolites, EsA was used as a positive control. First, we evaluated the effects of PPD, PPT, G-Rg3, G-Rh2, and EsA on eESCs and normal ESCs (nESCs) in vitro. As shown, treatment with PPD, PPT, G-Rh2, or EsA led to a significant decrease in eESC viability, especially at concentrations beyond 40 µM (Fig. 1b). Among these compounds, PPD has the most powerful inhibition on eESC viability, with an IC_50_ (half maximal inhibitory concentration) of 30.64 µM (Fig. 1c). However, none of these compounds (PPD, PPT, G-Rh2, or EsA) showed a significant effect on the viability of nESCs unless their concentrations were higher than 80 µM (Supplementary Figure 1). Further analysis showed that the expression levels of anti-apoptosis molecules B-cell lymphoma (Bcl)-xL and Bcl-2, and proliferation-related molecules Ki-67 and proliferating cell nuclear antigen (PCNA) in PPD-treated eESCs were obviously decreased compared with those of control eESC and EsA-treated eESCs (Fig. 1d, e). Conversely, the tumor metastasis suppressor CD82^25^ and pro-apoptotic molecules Bax and Bak were increased (Fig. 1d–f). These results indicated that a low level of PPD can markedly inhibits the viability of eESCs not nESCs in vitro.

### PPD suppresses estrogen-mediated autophagy inhibition of eESCs possibly by downregulating ERα and upregulating progesterone receptor α (PRα) in vitro

**Fig. 2:**
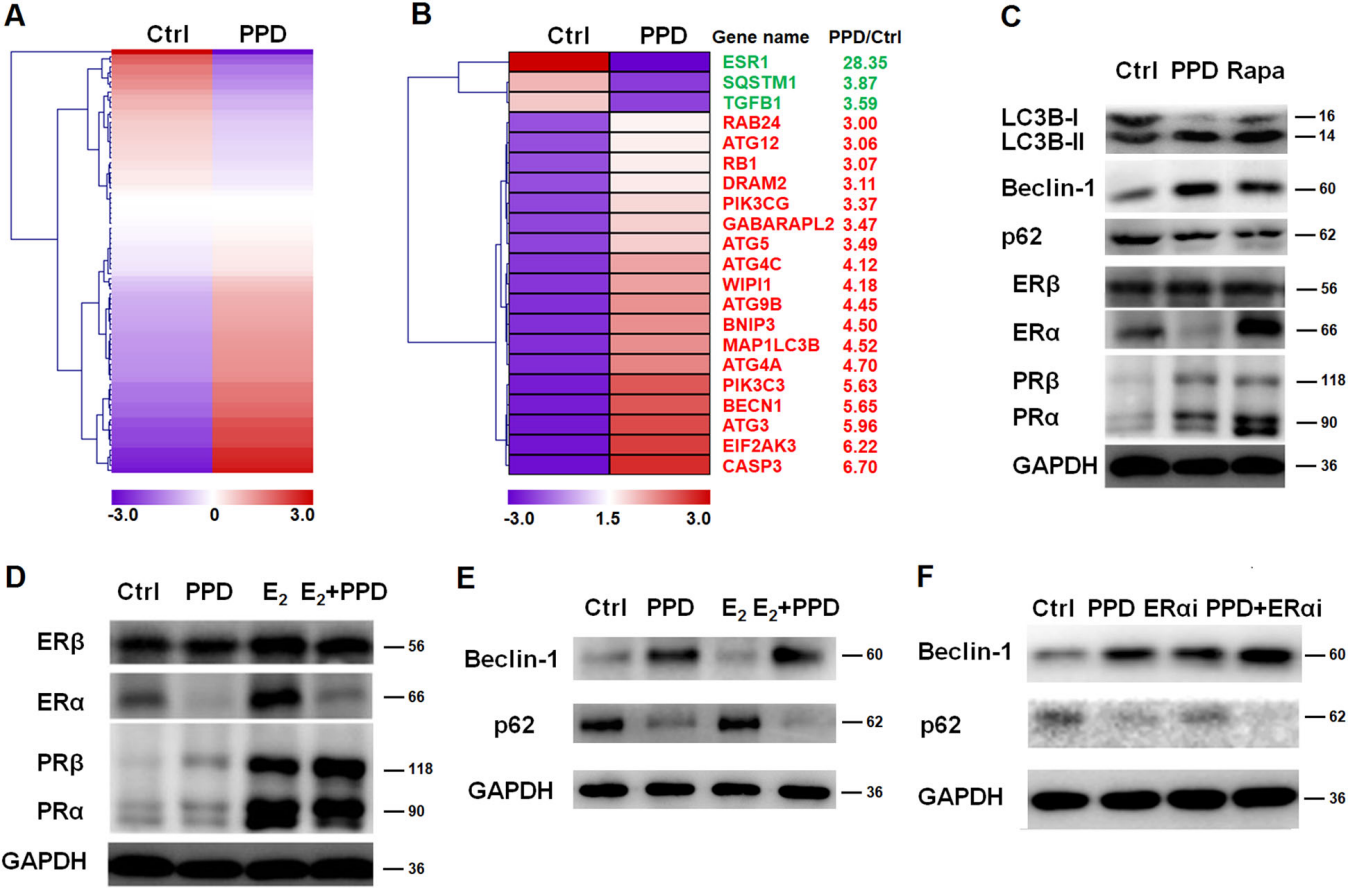
PPD suppresses the estrogen-mediated autophagy inhibition of eESCs possibly by downregulating ERα and upregulating progesterone receptor α (PRα) in vitro. **a** Human Autophagy RT^2^ profiler™ PCR array was performed to evaluate the differential expression of autophagy-related genes in eESCs after treatment with or without PPD (40 µM) for 48 h. **b** Twenty-one different genes between control eESCs and PPD-treated eESCs (fold difference >3 in eESCs). **c** The eESCs (*n* = 6) were indicated with PPD (40 µM) or rapamycin (1 µM) for 48 h, and then western blotting was used to analyze the expression levels of LC3B, Beclin-1, p62, ERα, ERβ, PRα, and PRβ in eESCs. **d**, **e** After treatment with PPD (40 µM), 17β-estrogen (E_2_, 10^−7^M) or PPD plus E_2_ for 48 h, the expression levels of ERα, ERβ, PRα, PRβ, Beclin-1, and p62 in eESCs (eESCs, *n* = 6) were analyzed by western blotting. **f** After treatment with PPD (40 µM), ERα antagonist (MPP dihydrochloride, 2.7 nM), or PPD plus MPP dihydrochloride for 48 h, the expression levels of Beclin-1 and p62 in eESCs (*n* = 6) were analyzed by western blotting. ERαi MPP dihydrochloride, Rapa rapamycin

To investigate the potential effect of PPD on the autophagy of eESCs, quantitative real time polymerase chain reaction (qRT-PCR)-based analysis of 84 genes involved in autophagy (a human autophagy PCR array) was performed (Fig. 2a). As shown, 21 different genes (such as autophagy-related genes *(ATG)2*, *ATG3*, and *ATG5*, *ESR1*, *SQSTEM1*, and *TGFB1*) between control eESCs and PPD-treated eESCs (the differential fold >3 in eESCs) was found (Fig. 2b). The results of western blotting showed that PPD upregulated autophagy-related molecules microtubule-associated protein light chain 3B (LC3B)-II, Beclin-1, and downregulated p62 expression in eESCs (Fig. 2c), suggesting that PPD could enhance the autophagy of eESCs.

Meanwhile, treatment with PPD led to a significant decrease in ERα and increase in PRα in eESCs (Fig. 2c). PPD could restrict the stimulatory effect on ERα expression and inhibitory effect on autophagy induced by 17-β estradiol (E_2_) in eESCs (Fig. 2d, e). In addition, PPD alone, ERα antagonist MPP dihydrochloride alone or the combination of PPD and MPP dihydrochloride resulted in the elevated autophagy of eESCs (Fig. 2f). In addition, MPP dihydrochloride led to a low level of Bcl-2/Bcl-XL and a high level of Bax/Bak, similar to that with PPD, but there was no synergistic effect with the combined treatment of MPP dihydrochloride plus PPD (Supplementary Figure 2). These data suggest that PPD can downregulate ERα expression and upregulate PRα expression, and further induce a high level of autophagy and high ratio of pro-apoptosis molecules (Bax/Bak) to anti-apoptosis molecules (Bcl-2/Bcl-XL) in eESCs in vitro.

### PPD activates NK cells in response to eESCs in a co-culture model

**Fig. 3:**
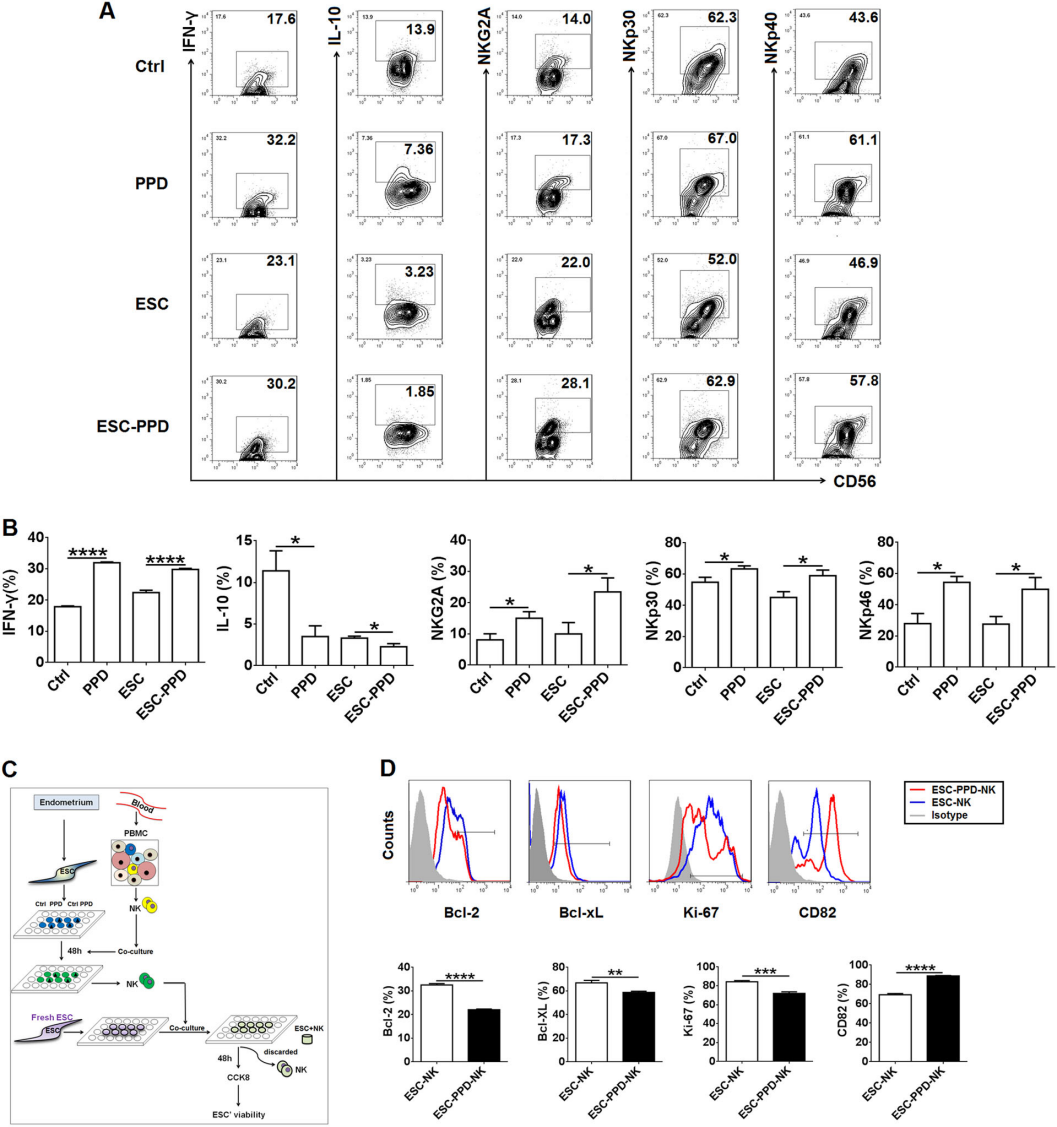
PPD activates NK cells in response to eESCs in a co-culture model. **a**, **b** NK cells isolated from PBMCs were treated with PPD or were co-cultured with control eESCs or PPD-pretreated eESCs (*n* = 6) for 48 h, and then the expression levels of IFN-γ, IL-10, NKG2A, NKp30, and NKp46 in NK cells were analyzed by FCM. Ctrl: NK cells alone; PPD: NK cells treated with PPD; ESC: NK cells co-cultured with control eESCs; ESC-PPD: NK cells co-cultured with PPD-pretreated eESCs (one-way ANOVA). **c**, **d** After co-culture with control eESCs (ESC-NK) or PPD-pretreated eESCs (ESC-PPD-NK) for 48 h, the NK cells were collected and further co-cultured with fresh eESCs (*n* = 6) for another 48 h. The expression levels of Bcl-2, Bcl-xL, Ki-67, and CD82 in eESCs were analyzed by FCM. (Student’s *t*-test). Data are expressed as mean ± SEM. *P < 0.05, **P < 0.01, ***P < 0.001, and ****P < 0.0001

To explore the potential role of PPD in NK cells, NK cells were isolated from the peripheral blood of healthy women and were directly treated with PPD, or co-cultured with control eESCs or PPD-pretreated eESCs. Next, we found that both PPD treatment and co-culture with PPD-pretreated eESCs increased the expression of natural killer group 2A (NKG2A) and activating natural cytotoxicity receptors (NKp30 and NKp46)^26,27^ and cytokine interferon (IFN)-γ, and decreased IL-10 expression in NK cells (Fig. 3a, b).

To further investigate the role of these NK cells in the viability of eESCs, we collected NK cells after co-culture with control eESCs (ESC-NK) or PPD-pretreated eESCs (ESC-PPD-NK), and then co-cultured with fresh eESCs (Fig. 3c). As shown, compared with ESC-NK co-cultured eESCs, the expression of Bcl-2, Bcl-xL, and Ki-67 was markedly decreased, and the expression of CD82 was increased in ESC-PPD-NK co-cultured eESCs (Fig. 3d). These data suggest that PPD can induce the activation directly and indirectly by acting on eESCs, and PPD-pretreated eESC-cultured NK cells may further restrict the growth and invasion in vitro.

### Low doses of PPD do not influence ERα or PRα expression, proliferation, or the autophagy of the uterine endometrium in a mouse EMS model

**Fig. 4:**
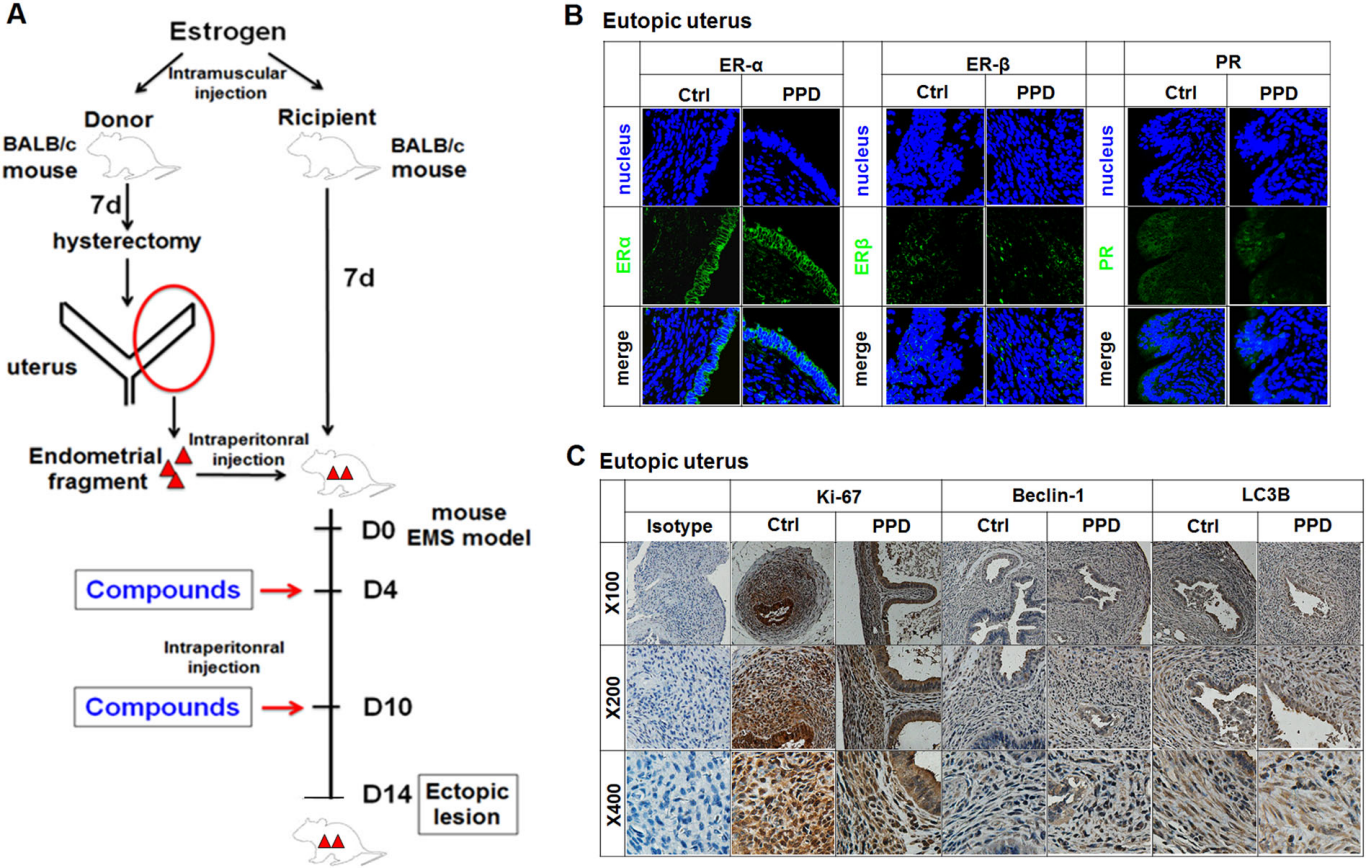
Low doses of PPD do not influence ERα or PRα expression, proliferation, or autophagy of the uterine endometrium in a mouse EMS model. **a**–**c** The EMS model of BALB/C mice was constructed by allotransplantation and was intraperitoneally injected with low doses of PPD (15 mg/kg, 100 µl) on day 4 and day 10. On day 14, the expression levels of ERα, ERβ, PR, Ki-67, Beclin-1, and LC3B in mouse uterine tissues were detected by immunofluorescence (IF, original magnification: ×200) (**b**) and immunohistochemistry (IHC) (**c**)

To evaluate the potential therapeutic value of PPD in EMS, we analyzed the role of PPD in eutopic endometrium. According to the procedure of Figure 4a, the mouse EMS model was constructed by allotransplantation, and was intraperitoneally injected with low doses of PPD. As shown, PPD did not change the expression of ERα, PRα, Ki-67, Beclin-1, and LC3B in mouse uterine endometrium (Fig. 4b, c), suggesting that intraperitoneal injection of low-dose PPD does not regulate ERα and PRα expression, proliferation, and autophagy of uterine endometrium in a mouse EMS model.

### PPD reverses the inhibitory effects of estrogen on the autophagy of mouse ectopic lesions and the activation of NK cells

**Fig. 5:**
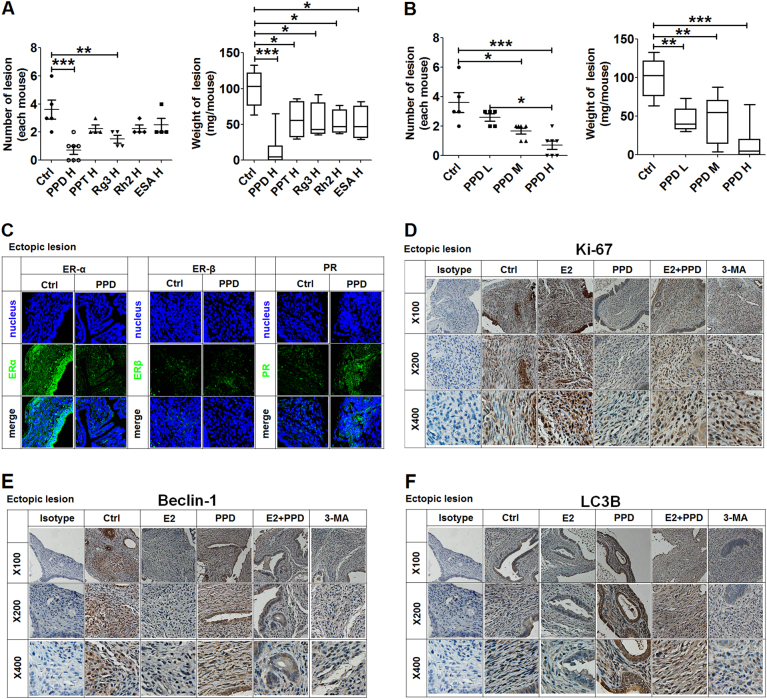
PPD reverses the inhibitory effects of estrogen on the autophagy of mouse ectopic lesions. **a** The number and weight of ectopic lesions in a mouse EMS model after intraperitoneal injection of the compounds (PPD, PPT, G-Rg3, G-Rh2, or EsA) at a high dose (45 mg/kg, 100 µl) (one-way ANOVA). **b** The number and weight of ectopic lesions in mouse EMS model after intraperitoneal injection of PPD at a high dose (PPD H, 45 mg/kg, 100 µl), medium dose (PPD M, 30 mg/kg, 100 µl) or low-dose (PPD L, 15 mg/kg, 100 µl) (one-way ANOVA). **c**–**f** The mouse EMS model was intraperitoneally injected with low doses of PPD (15 mg/kg, 100 µl), E_2_ (150 µg/kg,100 µl; intramuscular injection) and or PPD (15 mg/kg, 100 µl; intraperitoneal injection), or 3-MA (50 mg/kg, 100 µl; intraperitoneal injection) on day 4 and day 10. On day 14, the expression levels of ERα, ERβ, PR, Ki-67, Beclin-1, and LC3B in mouse ectopic lesions were detected by IF (original magnification: ×200) (**c**) and IHC (**d**–**f**)

As shown, all compounds (PPD, PPT, G-Rg3, G-Rh2, and EsA) at a high dose could significantly reduce the number and weight of ectopic lesions in a mouse EMS model, especially PPD (Fig. 5a). A significant inhibitory effect on the weight of mouse ectopic lesions could be achieved with the administration of low doses of PPD (Fig. 5b).

Subsequently, we found that PPD led to the decrease in ERα and the increase in PRα in ectopic lesions (Fig. 5c), echoing the results in vitro. E_2_ significantly promoted the proliferation of ectopic lesions, and this effect could be reversed by PPD (Fig. 5d). E_2_ suppressed the expression of Beclin-1 and LC3B of ectopic lesions as well as 3-methyladenine (3-MA, an autophagy inhibitor), and PPD also completely abrogated it (Fig. 5e, f).

**Fig. 6:**
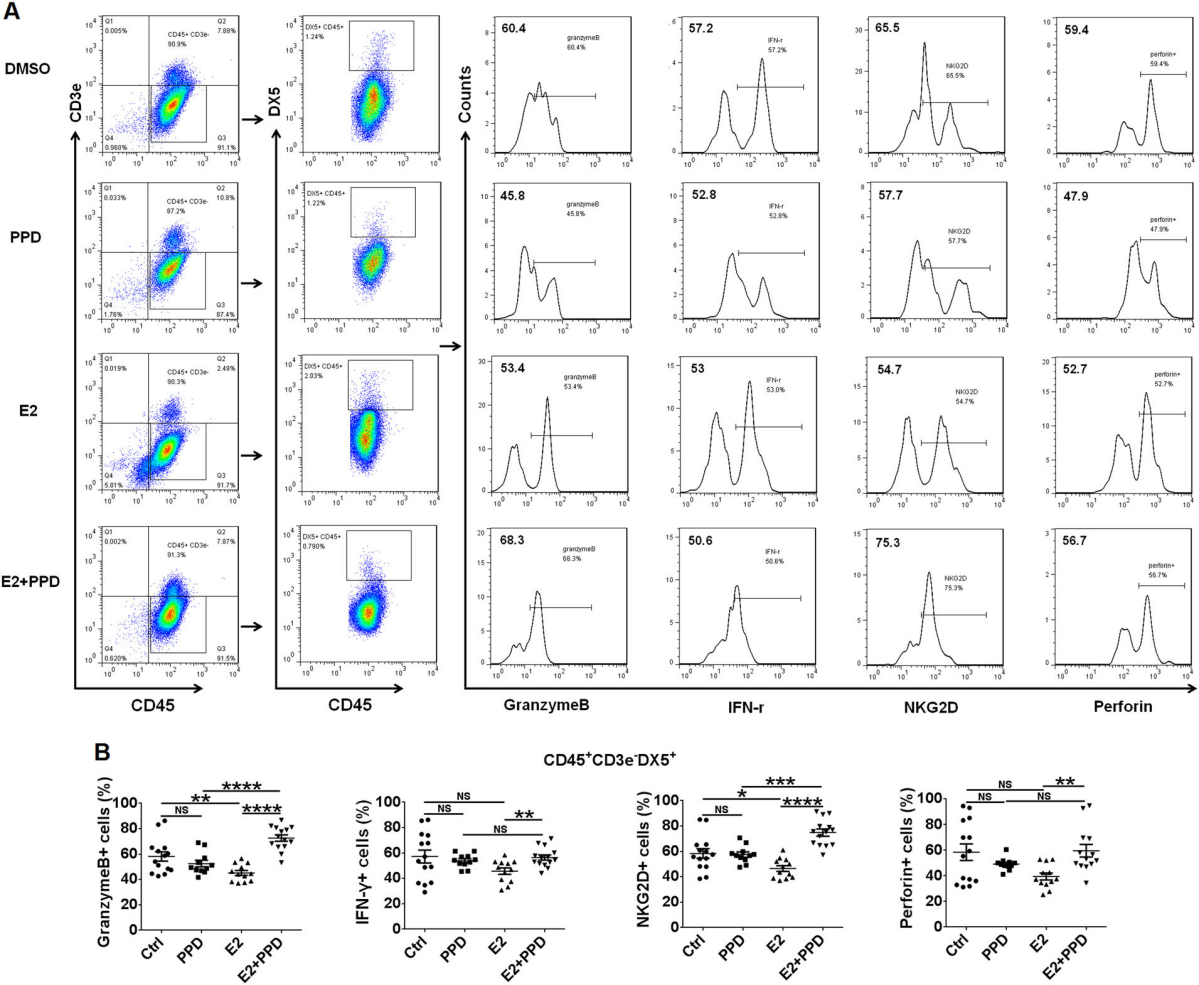
PPD reverses the inhibitory effects of estrogen on the activation of NK cells. **a**, **b** The mouse EMS model was intraperitoneally injected with low doses of PPD, E_2_ and/or PPD, or 3-MA on day 4 and day 10. On day 14, the expression levels of Granzyme B, NKG2D, IFN-γ, and perforin in CD45^+^CD3e^-^DX5^+^NK cells from mouse peritoneal fluid were analyzed by FCM (one-way ANOVA). Data are expressed as mean ± SEM. *P < 0.05, **P < 0.01, ***P < 0.001, and ****P < 0.0001

In addition, treatment with E_2_ downregulated Granzyme B and NKG2D in CD45^+^CD3e^-^DX5^+^NK cells from mouse peritoneal fluids (Fig. 6a, b). Although the intraperitoneal injection of PPD had no similarly effects on NK cells, the combination of PPD and E_2_ induced the activation of NK cells compared with E_2_ alone (Fig. 6a, b).

### PPD suppresses the estrogen-mediated growth of mouse ectopic lesions

**Fig. 7:**
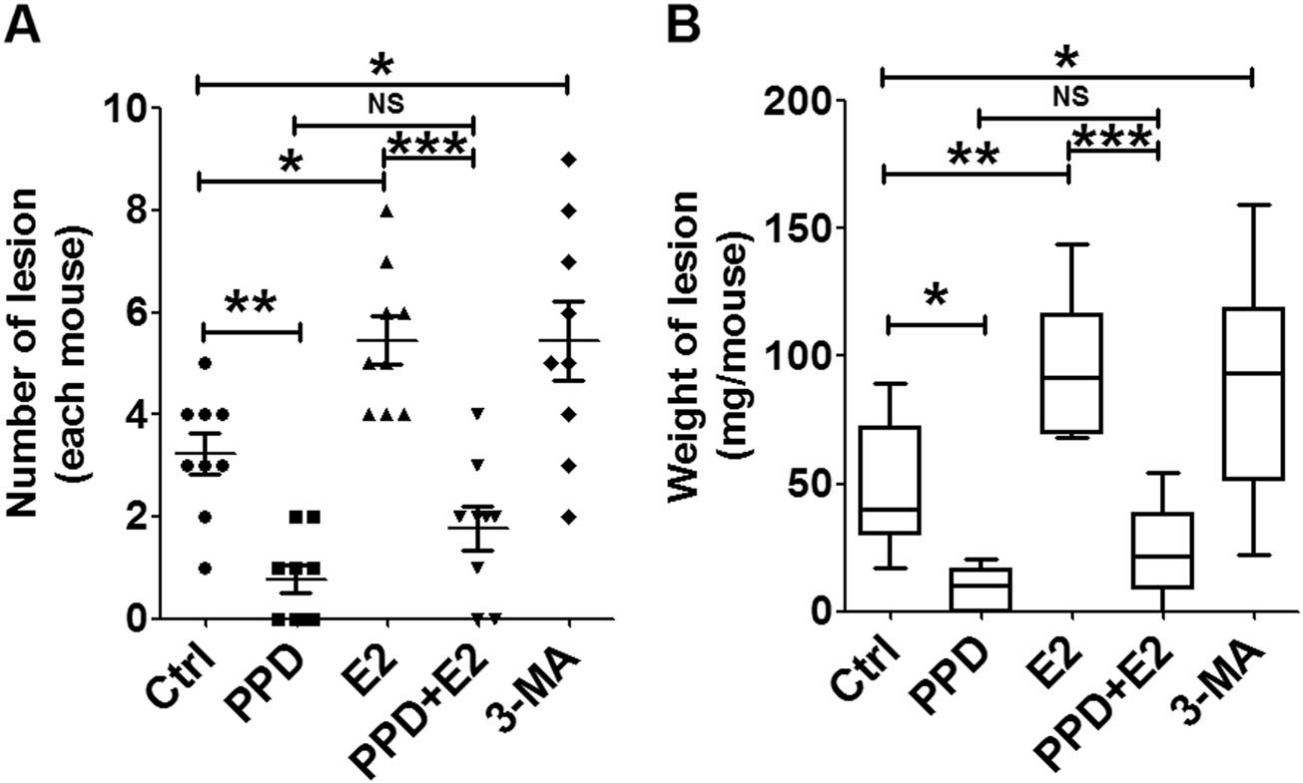
PPD suppresses the estrogen-mediated growth of mouse ectopic lesions. **a**, **b** Number and weight of ectopic lesions from the mouse EMS model, which was treated as described in Figure 6. Data are expressed as mean ± SEM. *P < 0.05, **P < 0.01, and ***P < 0.001

Finally, we analyzed the effect of PPD and E_2_ on the number and weight of ectopic lesions in a mouse EMS model. As shown, E_2_ increased the number and weight of mouse ectopic lesions (Fig. 7a, b). In contrast, PPD not only decreased the number and weight of mouse ectopic lesions, but also restricted the stimulatory effect of E_2_ on the growth of mouse ectopic lesions (Fig. 7a, b). These data suggest that PPD suppresses the growth of mouse ectopic lesions induced by estrogen and plays a role in anti-mouse EMS activity.

## Discussion

Ginsenosides within the dammarane-type consist mainly of three types classified according to their genuine aglycone moieties: PPD, PPT, and ocotillol. The potential health effects of ginsenosides include anti-inflammatory, antistress, anticarcinogenic, immunomodulatory, antiallergic, antiatherosclerotic, and antihypertensive effects as well as antidiabetic effects and regulatory effects on blood pressure and metabolism^28–30^. Here, we found that PPD, PPT, G-Rg3, and G-Rh2, especially PPD, could inhibit viability and growth as well as EsA both in vitro and in vivo. Additionally, low doses of PPD did not influence the growth of nESCs or the eutopic endometrium. These results suggest that PPD may be a safe and effective strategy for the treatment of EMS.

Autophagy has been linked to various pathophysiological processes, including cell death, development, tumorigenesis, and immunity^31,32^. We had found that autophagy in eESCs was significantly decreased, and estrogen suppressed the autophagy of ESCs by upregulating CXCL12 and CXCR4 expression. Here, the analysis of autophagy-related genes in this study showed that PPD enhanced the transcription of several autophagy-related genes in eESCs, such as *ATGs* and *MAP1L3CB*. Of note, the *ESR1* and *TGFB* mRNA levels were decreased. Under hormonal control, TGF-β is abundantly and differentially expressed in the endometrium and is involved in the promotion of ESC survival, cell adhesion and invasion, angiogenesis^33^. Additionally, TGF-β contributes to the suppression of the immune system in the environment of ectopic lesions such as the induction of IL-10^+^T help (Th)17 and regulatory T (Treg) cell differentiation and the impairment of NK cells cytotoxicity^9,33–35^. Therefore, the anti-EMS effects of PPD may also be dependent on the regulation of *TGFB*.

Estrogen and progestogen modulate proliferation, apoptosis, and autophagy in the human endometrium and in endometriotic cells and tissues, further contributing to the origin and development of EMS^7,36^. In the current study, we also observed that E2 treatment led to increased Ki-67 expression and a low autophagy level in ectopic lesions and elevated numbers and weights of ectopic lesions in a mouse EMS model. In addition, studies using ER knockout transgenic mice and treatment with ER subtype-selective ligands have indicated the crucial roles of ERs in EMS^37-40^. Progesterone resistance was observed in EMS. The molecular basis of progesterone resistance in EMS may be related to an overall reduction in the levels of PR^37^. The progesterone responses are mediated primarily via binding to and activation of the nuclear receptors (PR-A and PR-B)^38,41^. We found that PPD not only decreased ERα expression but also upregulated PRα expression in eESCs in vitro and in vivo. Our previous works have reported that progesterone signaling can suppress the inhibitory effect of estrogen on ESC autophagy^7^. Therefore, the stimulation of PR likely plays an important role in anti-EMS by PPD.

Here, we observed that PPD upregulated Bax and Bak, and downregulated Bcl-2 and Bcl-xL in ectopic ESCs possibly by downregulating ERα. In addition to ER signaling, other signaling pathways such as PI3K/AKT, ROS and NF-κB participate in the regulation process of ginsenosides in cell apoptosis and death^42–45^. Under the regulation of estrogen, these signaling pathways are also involved in regulating ESC’s apoptosis in EMS^36,46–48^. Therefore, PPD may also regulate these apoptosis-related proteins via these signaling pathways (e.g., PI3K/AKT, ROS, and NF-κB) in EMS, a finding that require further research.

Ginsenosides are structurally described as triterpenoid saponins that contain a steroidal backbone. Although most naturally occurring ginsenosides have bulky sugar side chains that pose a huge steric hindrance for these molecules to bind to steroid receptors, various functional assays and molecular docking studies have provided evidence to show that ginsenosides can mediate their cellular activities by binding to the active sites of steroid receptors and downregulating the expression of estrogen receptors^20,22,49^. On the other hand, other studies suggest that ginsenosides may modulate their cellular actions via pathways independent of steroid receptors^50–52^. However, the regulatory mechanism of ginsenosides for hormone receptors remain unclear and warrants further research.

The impaired cytotoxic activity of NK cells is associated with several physiological and pathological processes, including EMS^14,27,53^. The effector functions of NK cells include the cytotoxicity and secretion of cytokines (e.g., IFN-γ, TNF-α). The most potent activated receptors of NK cells are the antibody-dependent cell-mediated cytotoxicity (ADCC)-mediating molecule CD16 and NKG2D^26,27^. Moreover, NK cells recognize their ligands in tumor or virus-infected cells and mediate natural cytotoxicity through a set of activating natural cytotoxicity receptors (e.g., NKp30, and NKp46)^26,27^. In the current study, PPD promoted the cytotoxic activity of NK cells, such as via the upregulation of granzyme B and perforin, especially under the stimulation of estrogen. Of note, we did not observe the effect of PPD alone on the activation NK cells in the mouse EMS model. This result may have resulted from the low concentration and or complex microenvironment in EMS, but the mechanisms deserve further investigation.

EMS is also an inflammatory disorder. The action of estrogen not only is essential for endometriotic tissue growth but also contributes to ongoing inflammation, neovascularization, and associated pain^54^. Ginsenosides are known to have anti-cancer properties based on their anti-inflammatory activation, and their low toxicities render them excellent candidates for cancer therapy^20^. For example, ginseng total saponins markedly reduce the production of pro-inflammatory cytokines (e.g., TNF-α, interleukin (IL)-1β, and IL-6) in lipopolysaccharide (LPS)-stimulated rat astrocyte and microglia cultures in vitro. These inflammatory cytokines were also increased in the eESCs, ectopic lesions and peritoneal fluids from patients with EMS^40,55^. Therefore, the anti-EMS property may be partly dependent on the anti-inflammatory effects, which requires further research.

**Fig. 8:**
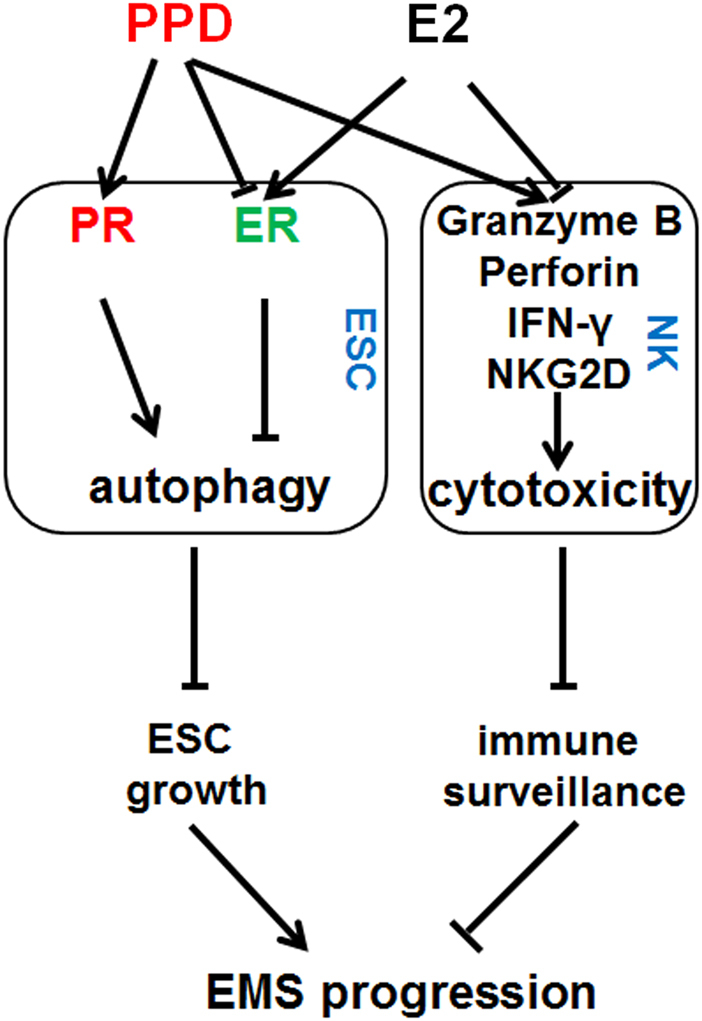
Schematic roles of ginsenoside PPD in anti-EMS activation. PPD can downregulate the expression of ERα and upregulate the expression of PRα. The inhibition of estrogen/ERα signaling mediated by PPD further induces the autophagy of eESCs and activates the cytotoxicity of NK cells (upregulation of granzyme B, NKG2D, IFN-γ, and perforin) in response to eESCs, and suppresses the growth of ectopic lesions and enhances the immune surveillance to ectopic lesions finally inhibiting the development of EMS

Collectively, our findings, schematized in Figure 8, reveal that PPD can downregulate the expression of ERα and upregulate the expression of PRα. The inhibition of estrogen/ERα signaling mediated by PPD further induces the autophagy of eESCs, activates the cytotoxicity of NK cells in response to eESCs, suppresses the growth of ectopic lesions, enhances the immune surveillance of ectopic lesions, and finally inhibits the development of EMS. Therefore, PPD should have potential therapeutic value for treating diseases such as EMS. Differences in the saponin structure markedly influence bioactivity and bioavailability. Therefore, further structural modification of PPD is needed to identify better anti-EMS drugs.

## Materials and methods

### Patients and sample collection

The study protocol was approved by the Human Research Ethics Committee of Obstetrics and Gynecology Hospital, Fudan University, and written informed consent was obtained from all patients. All of the endometriotic tissues were obtained by laparoscopy from 43 patients with EMS at the Obstetrics and Gynecology Hospital of Fudan University. Normal endometrium was obtained through hysterectomy from patients with leiomyoma (six cases) as healthy control samples. None of the included patient experienced complications related to pelvic inflammatory disease and no patient took any medication or received hormonal therapy within six months prior to surgery. All the samples were obtained in the proliferation phase of the cell cycle, as confirmed histologically according to established criteria.

### Isolation and culture of ESCs

We isolated human nESCs from the endometrium of healthy control subjects, and eESCs from ectopic lesions of women with EMS according to a previously described method^25^. It supplied >98% vimentin^+^CK7^-^ ESCs, as confirmed by flow cytometry (FCM) analysis.

### Purification of NK cells

Peripheral blood mononuclear cells (PBMCs) were isolated from healthy fertile women. Human NK cells were isolated from PBMCs using magnetic beads (Miltenyi Biotec, Bergisch Gladbach, Germany) for in vitro experiments. These NK cells were directly treated with PPD (40 µM, Sigma-Aldrich Co. LLC., USA); or were co-cultured with control eESCs or PPD-pretreated eESCs for 48 h, and then these NK cells were collected to analyze the expression of IFN-γ, IL-10, NKG2A, NKp30, and NKp40 by FCM; or were further co-cultured with fresh eESCs for another 48 h, and then the expression levels of Bcl-2, Bcl-xL, Ki-67, and CD82 in eESCs were analyzed by FCM.

### The cell-counting kit-8 (CCK-8) assay

The eESCs were treated with PPD, PPT, G-Rg3, G-Rh2, or EsA (0–160 µM, Sigma-Aldrich Co. LLC., USA) for 24 or 48 h, with 0.1% DMSO as a blank control. Next, these cells were collected, and the viability was detected by the CCK-8 assay (Dojindo, Japan). According to the manufacturer’s protocol, the CCK-8 reagent was added to each well and cells were incubated at 37°C for 1–4 h. The absorbance (optical density) at 450 nm was measured and used to represent the cell viability. Each experiment was performed in six parallel wells and repeated three times.

### FCM

After treatment with PPD (40 µM) or EsA (40 µM), or co-culture with control eESCs (ESC-NK) or PPD-pretreated eESCs (ESC-PPD-NK), these eESCs were collected and the expression levels of Bcl-2, Bcl-xL, Bax, Ki-67, PCNA, and or CD82 (all from Biolegend, USA) were analyzed by FCM according to the manufacturer’s instructions. In addition, the expression levels of IFN-γ, IL-10, NKG2A, NKp30, and NKp40 (all from Biolgend) in CD56^+^NK cells were analyzed by FCM. Isotypic IgG antibodies were used as controls. The samples were analyzed using a FACS-Calibur flow cytometer (Becton Dickinson, USA) and Cellquest software (Becton Dickinson). Statistical analysis was conducted using isotype-matched controls as references.

### Human Autophagy RT^2^ profiler™ PCR array

The eESCs were indicated with PPD (40 µM) for 48 h, with 0.1% DMSO as a blank control. Next, human Autophagy RT^2^ profiler™ PCR array (Catalog No. PAHS-084ZR, 96-well format) was utilized to analyze the transcriptional levels of these autophagy-related genes in eESCs as described previously^7,56^. The plates were processed using an Applied Biosystems 7500 fast RT-PCR system (Applied Biosystems, USA), and the data were interpreted with SABiosciences’ web-based PCR array analysis tool.

### Protein extraction and western blotting

The eESCs were treated with PPD (40 µM), rapamycin (1 µM, sigma), E2 (10-^7^M, Sigma), E2 plus PPD, ERα antagonist (MPP dihydrochloride; 2.7 nM; Tocris Bioscience, USA), or PPD plus MPP dihydrochloride for 48 h, and then cells were washed in phosphate buffered saline (PBS), detached with a cell scraper and centrifuged for 20 min at 12,000 r.p.m. at 4 °C. The pellet was resuspended in high efficiency cell tissue rapid lysis buffer (RIPA; Beyotime, Shanghai, China) containing 1% phenylmethanesulfonylfluoride (PMSF; Beyotime) proteinase and 1% phosphatase inhibitors (Roche, USA). Cell lysates were boiled for 10 min at 95 °C and then were stored at −80 °C. Protein concentrations were quantified using the BCA protein assay kit (Beyotime). Total proteins (20 µg) were electrophoresed in SDS-PAGE gels (EpiZyme scientific) using a Miniprotein III system (Bio-Rad, USA) and were transferred to PVDF membranes (Millipore, USA) at 2 h, followed by overnight incubation with primary antibody against Bax, Bak, Beclin-1, p62, LC3B, ERα, PR, or GAPDH (1:1000; Cell Signaling Technology, USA), and ERβ (1:200; Santa Cruz Biotechnology, Santa Cruz, CA, USA) at 4 °C. Then PVDF membranes were washed three times with PBST solution and were incubated at room temperature for 1 h in peroxidase-conjugated goat anti-rabbit IgG secondary antibodies (1:5000; Bioworld Technology, Co. Ltd. USA). Thereafter the membrane was washed three times and processed for chemiluminescence using the Immobilon Western Chemiluminescent HRP Substrate Kit (Millipore).

### Mouse EMS model

A group of adult female BALB/C mice was purchased from the Laboratory Animal Facility of Fudan University and was used for this study. They were maintained for 2 weeks at the animal facility before use. The Animal Care and Use Committee of Shanghai First Maternity and Infant Hospital, Tongji University School of Medicine approved all the animal protocols.

We constructed an intraperitoneal EMS model. On Day 0, the uterus of female BALB/C mice (Donor mice) was minced, and then the tissue debris was intraperitoneally injected into female BALB/C mice (for recipient mice, the ratio of the uterus to intraperitoneal injection of mice was 1:2). On day 4 and day 10, the EMS mice were intraperitoneally injected with the compounds (PPD, PPT, G-Rg3, G-Rh2, or EsA) at a high dose (45 mg/kg, 100 µl), medium dose (30 mg/kg, 100 µl) or low-dose (15 mg/kg, 100 µl). In addition, some EMS mice were treated with E_2_ (150 µg/kg,100 µl; intramuscular injection), and/or PPD (15 mg/kg, 100 µl; intraperitoneally injection), or 3-MA (50 mg/kg, 100 µl; intraperitoneally injection), 0.1% DMSO (100 µl) as a control. On Day 14, the EMS-like lesions and peritoneal fluids were collected and detected. The number and weight of EMS-like lesions were counted. Immunohistochemistry (IHC) and immunofluorescence (IF) were used to analyze the expression of ERα, ERβ, PR, Ki-67, Beclin-1 and LC3B in the EMS-like lesions. Additionally, the expression levels of IFN-γ, NKG2D, granzyme B and perforin (all from Biolegend) in CD45^+^CD3e^-^DX5^+^NK cells in peritoneal fluid were analyzed by FCM.

### IHC and IF

For IHC, paraffin sections (5 µm) of the EMS-like lesions and uterine endometrium were dehydrated in graded ethanol and then were incubated with hydrogen peroxide and 1% bovine serum albumin/TBS to block endogenous peroxidase. The samples were then incubated with rabbit anti-human Ki-67 (1:500; Abcam, USA), Beclin-1 (1:200; Abcam) and LC3B (1:500; Abcam) or rabbit IgG isotype (Abcam) overnight at 4 °C in a humid chamber. After washing three times with TBS, the sections were overlaid with peroxidase-conjugated goat anti-rabbit IgG, and the reaction was developed with 3,3-diaminobenzidine (DAB) and counterstained with hematoxylin.

For IF, according to a previous procedure^7^, EMS-like lesions and uterine endometrium tissues were incubated with anti-goat ERα, anti-goat ERβ, anti-goat PR antibody (1:100, Abcam) in PBS at 4 °C overnight. The slides were then incubated with Alexa Fluor 488-conjugated donkey anti-goat secondary antibody (1:500; Abcam). The nuclei were then stained with 4′,6-diamidino-2-phenylindole (DAPI; Beyotime, China). Images were captured with a confocal microscope (Leica, Germany).

### Statistics

All values are shown as the mean ± SEM. The data were analyzed with GraphPad Prism version 5 by *t*-test for two groups or one-way analysis of variance using Tukey’s post hoc test for multiple groups. Differences were considered statistically significant at *P* < 0.05.

## Electronic supplementary material

supplementary information

Supplmentary Figure 1

Supplmentary Figure 2
