# Supplementary material for: The ginsenoside PPD exerts anti-endometriosis effects by suppressing estrogen receptor-mediated inhibition of endometrial stromal cell autophagy and NK cell cytotoxicity
Source: Cell Death Dis. 2018 May 14;9(5):574. doi: 10.1038/s41419-018-0581-2 (PMC5951853; doi:10.1038/s41419-018-0581-2)
Supplement: Supplementary file 1 — supplementary information [file 41419_2018_581_MOESM1_ESM.doc]

**Supplementary Information**

**Supplementary Figure legends**

**Supplementary Figure 1: The effects of PPD, PPT, G-Rg3, G-Rh2 or EsA on normal ESCs.** After treatment with PPD, PPT, G-Rg3, G-Rh2 or EsA at different concentrations (0-160uM) for 24 or 48h, the viability of normal ESCs (n=6) was analyzed by CCK-8 assay. Data are expressed as mean±SEM. **P*<0.05, ***P*<0.01 and ****P*<0.001. **(**One-way ANOVA)

**Supplementary Figure 2:** **PPD regulates apoptosis-related molecules possibly by down-regulating ERα.** After treatment with PPD (40uM), ERα antagonist (MPP dihydrochloride, 2.7nM) or PPD plus MPP dihydrochloride for 48h, the expression of Bcl-xL, Bcl-2, Bax and Bak in eESCs (n=6) was analyzed by western blotting. ERαi: MPP dihydrochloride. Data are expressed as mean±SEM.
