# Supplementary figures and images for: The ginsenoside PPD exerts anti-endometriosis effects by suppressing estrogen receptor-mediated inhibition of endometrial stromal cell autophagy and NK cell cytotoxicity
Source: Cell Death Dis. 2018 May 14;9(5):574. doi: 10.1038/s41419-018-0581-2 (PMC5951853; doi:10.1038/s41419-018-0581-2)

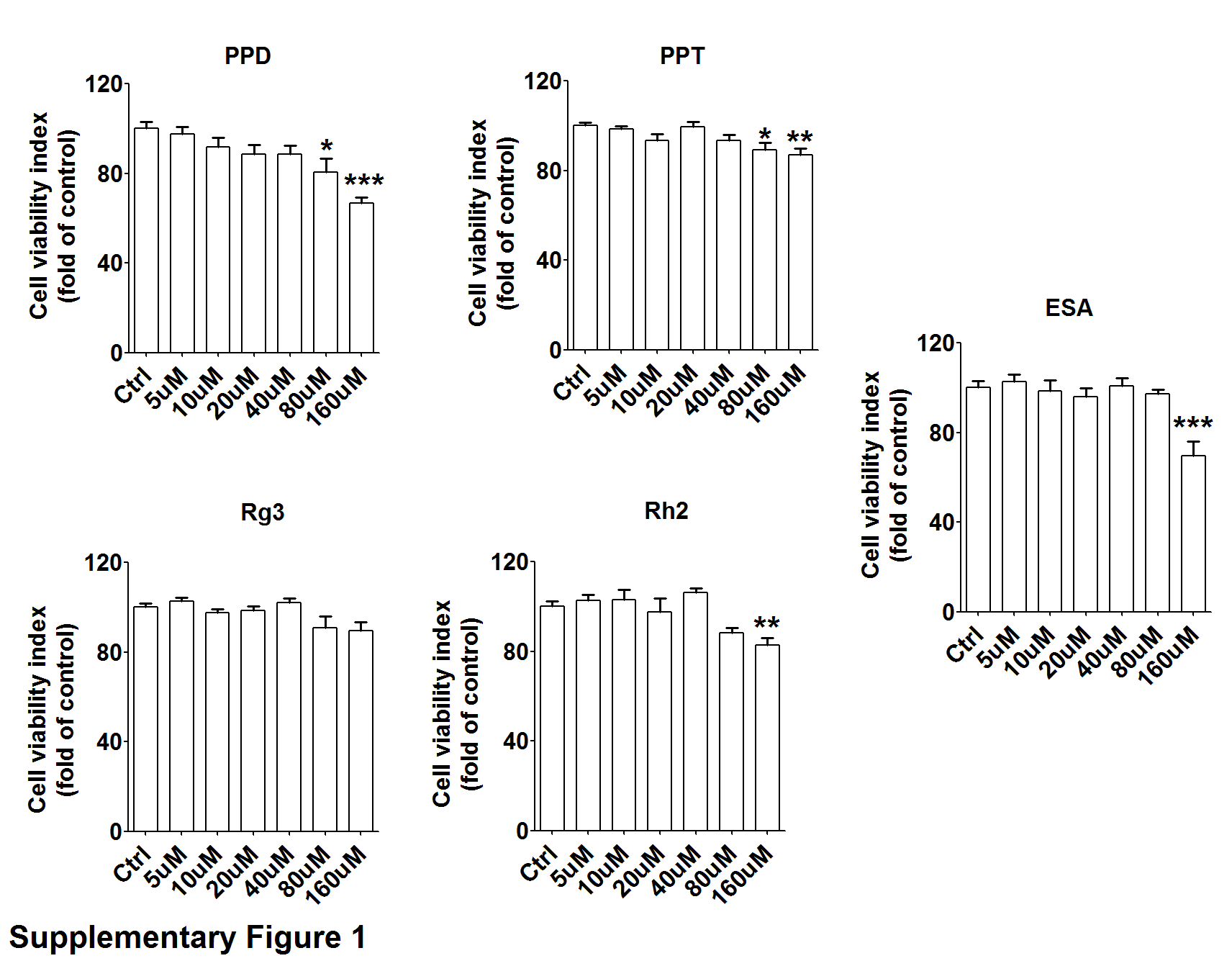

Supplement: Supplementary file 2 — Supplmentary Figure 1 [file 41419_2018_581_MOESM2_ESM.tif]

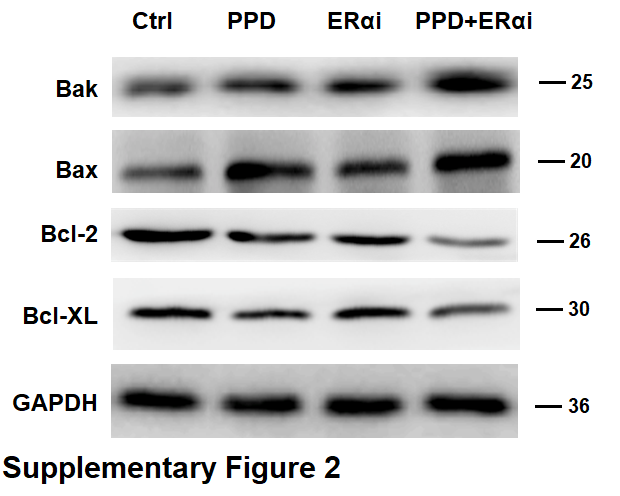

Supplement: Supplementary file 3 — Supplmentary Figure 2 [file 41419_2018_581_MOESM3_ESM.tif]
